# Supplementary figures and images for: Genomic and virulence analysis of in vitro cultured Cryptosporidium parvum
Source: PLoS Pathog. 2024 Feb 28;20(2):e1011992. doi: 10.1371/journal.ppat.1011992 (PMC10927135; doi:10.1371/journal.ppat.1011992)

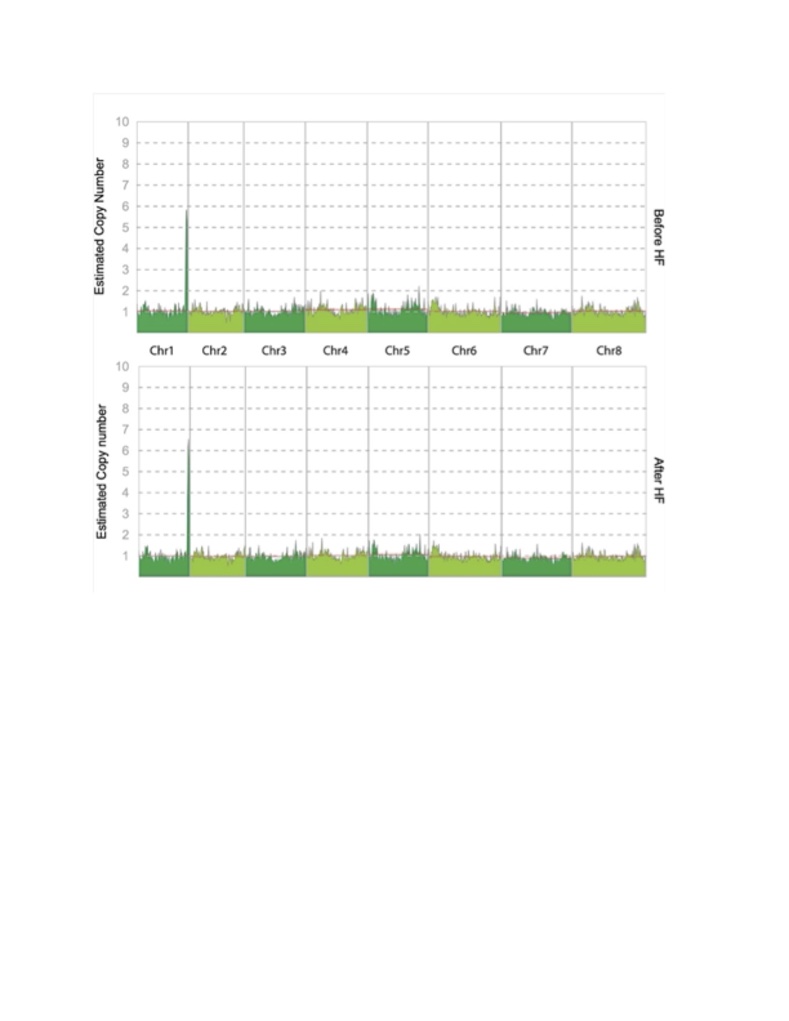

Supplement: S1 Fig — Sequence reads were aligned to C. parvum IOWA-ATCC genome assembly to assess deletions, insertions, and duplications. Both sample read depths were normalized by the whole genome average depth to get the estimated copy number across all chromosomes. (JPG) [file ppat.1011992.s001.jpg]
