# Supplementary material for: Genomic and virulence analysis of in vitro cultured Cryptosporidium parvum
Source: PLoS Pathog. 2024 Feb 28;20(2):e1011992. doi: 10.1371/journal.ppat.1011992 (PMC10927135; doi:10.1371/journal.ppat.1011992)
Supplement: Attachment — Submitted filename: Response to review (2).pdf [file ppat.1011992.s005.pdf]

The authors would like to thank the referees for the time and effort in reviewing the submitted manuscript. We have made the following edits based upon their comments:

Reviewer #1: In the discussion, page 15 line one the correct reference for in vitro production of *P. falciparum* sporozoites is [42] not [43].

Response: This has been changed as suggested.

Reviewer #2: Please see the comments in the attached PDF. Note I have made comments in the "Revised article with changes highlighted" version of the manuscript at the end of the file.

Specifically, the two most critical issues that need to be addressed are:
1. Revision of Figs 3A and 3B to ensure total fecal volumes are consistent.
2. Revision of the Fig 3C legend to align with the changes in the main text describing the Fig 3C results.

Response:

1. The authors are very grateful to reviewer 2 for pointing out this error. Fig 3A was correct however Fig 3B presented the total fecal volume over the 10 days for all animals in a group, however on revision the numbering was inadvertently listed as 1-10 L instead of 10-100 L, hence for BGF-T20HF it is approx. 18L/calf, which represents 72L total for the 4 calves in the group. On reconsidering how best to present the data we have changed Fig 3B to show the average total fecal volume from days 3-10 for a single calf $\pm$ SD of that group. We apologize for the confusion and hope the revised figure improves the presentation of the data.

2. We have revised the legend to Fig 3C to align with the main text.

Reviewer #3: No further comments

Response: None required.
