# Supplementary material for: Genomic and virulence analysis of in vitro cultured Cryptosporidium parvum
Source: PLoS Pathog. 2024 Feb 28;20(2):e1011992. doi: 10.1371/journal.ppat.1011992 (PMC10927135; doi:10.1371/journal.ppat.1011992)
Supplement: Attachment — Submitted filename: Response to PLoS Path review.docx [file ppat.1011992.s003.docx]

The authors thank the Editors and referees for their comments on the manuscript which improves the clarity.

**Editorial comments:**

i) Please give more details of the sequencing protocol use (for example only short reads or also long reads) and the depth of coverage obtain for each condition.

Response: We have added additional information concerning the sequencing protocol employed in the study on page 8 in the Methods section, under the sub-heading ‘Sequencing protocol’.

ii) Please indicate if there is a statistical difference in the amount of oocysts shed in the different infected groups when the data for the three groups is tested (perhaps a Kruskal Wallis test) or if the statistical difference only is evident when individual groups are compared with each other. It appears that the pattern of shedding between BGFT20 and BGF-2017 is very similar and that the peak shedding of Iowa is latter.

Response: We have analyzed the data as requested using a Kruskal Wallis test and included the analysis on page 12, line 4 of the Results section under the sub-heading ‘Calf infections’. The authors appreciate this comment as it supports the conclusions reached in the study.

iii) Please correct graph 3C (or text) as the number of days in the text differs from the number of data points shown in the graph.

Response: The abscissa has been corrected on Fig 3C which now corresponds with the correct day PI. The authors apologize for this error caused by switching to a new graphing program; and would like to thank the editors and referees for bringing this to our attention.

**Reviewer #1**: The others cite unpublished data on *T. gondii* in the culture system. If a manuscript has been submitted to a preprint service such as bioRxiv then this should be provided in the references.

Response: As of this time we are completing the data and have not submitted a manuscript. For proof of concept in a culture manuscript we typically monitor growth for 3-6 months, which we have successfully completed as of the end of August, 2023 and will be preparing a manuscript ASAP. The authors understand that unpublished data lacks the rigor of a published paper hence we have removed the statement from the Discussion.

**Reviewer #2**: Page 5, line 17 (Introduction); The statement that NTZ is inadequate in children under 3 years old is inaccurate. Reference #5 (Amadi et al. 2002 Lancet) found NTZ was modestly effective in HIV-negative children, but not HIV-positive children. I suggest instead highlighting here that NTZ is not approved for use in children under 12 months of age, which is the most population most vulnerable to the effects of cryptosporidiosis.

Response: This statement has been amended as suggested; page 3, line 16.

Page 13, line 14 (Calf infections); The sentence "The daily oocyst numbers shed indicate that BGF-T20HF is statistically significant compared to BGF-2017 and IOWA-2017 for days 3 and 10 PI" is confusing and needs to be reworded. It is unclear if the authors mean to say oocyst shedding from calves infected with BGF-T20HF is statistically significantly greater or less than BGF-2017 and IOWA-2017. Furthermore, in Figure 3C, it appears that BGF-T20HF is virtually identical to BGF-2017 on day 3 PI, so it is hard to understand how these values could be statistically significantly different. Finally, there is no data shown for day 10 PI, despite this data being referenced in the text and the figure legend.

Response: The authors sincerely apologize, there were several misleading statements and an error on axis of Fig 3C in the section which have now been thoroughly revised in the revised submission; page 12.

Page 13, line 15 (Calf infections); The sentence "All other days are significantly higher for BGF-T20HF (days 5-9 PI) and IOWA-2017 (days 4-9 PI) compared to BGF-2017 (Fig 3C)" is not consistent with the data shown in Fig 3C. For example, on day 8 PI, BGF-2017 appears to be virtually identical to IOWA-2017. Furthermore, on day 9 PI, BGF-2017 is greater than BGF-T20HF, contrary to what is stated.

Response: The authors agree and have revised these to be consistent with the data in Fig 3C.

Page 14, line 1 (Calf infections); Interpretation of the fecal consistency data (and reference to Fig 3D where it is shown) are missing. I suggest moving text from figure legend to the main body here (see comment in Fig 3D legend).

Response: This is an excellent suggestion and we have moved the information from the legend to the text; page 12, line 18.

Page 14, line 3 (Calf infections); The explanation "Onset of diarrhea was delayed in one BGF-T20HF infected calf (Fig 3F)" appears to be superfluous because the data in Figs 3E-F appear to be very similar for BGF-T20HF as compared to BGF-2017 and IOWA-2017.

Response: The authors agree with the reviewer and have amended the sentence accordingly; bottom of page 12-top of page 13. We also felt the sentence did not fully convey the data from this important result and have expanded slightly the text concerning Figs 3E and F. We thank the reviewer for this helpful suggestion.

Page 16, line 22 (Discussion); The reference after the sentence, "Parasites generated using this method...have great promise for a malaria vaccine" should be reference #43 (Eappen et al. 2022 Nature), not reference #44.

Response: corrected.

Page 27, line 12 (Figure 1 legend); Should be "HCT-8 cells", not simply "HCT cells".

Response: corrected.

Page 28, line 10 (Figure 3 legend); I suggest that the two interpretive sentences ("One calf infected...due to this delay.") are moved from the figure legend to the main body of the text where Fig 3D is described, as suggested above.

Response: corrected.

Page 30 (Figure 2); IOWA-2017 is referenced in the figure legend and elsewhere in the manuscript, but not shown in the figure. I recommend clarifying where IOWA-2017 fits in this schematic.

Response: The genome sequence information for *C. parvum* in the database was generated using the IOWA isolate, hence this isolate from a comparable date was included in the study to determine if there was marked differences between this isolate and the BGF isolate that was used to initiate the in vitro culture. Fig 2, is meant to show the culture path that produced BGF-T20HF, hence IOWA isolate is not shown.

Page 31 (Figure 3); The y-axis of Fig 3B is confusing because it uses scientific notation (e.g., "1e+4"), yet the scale is linear, not logarithmic (unlike Fig 3C, which is on a log scale). I suggest using standard notation for the y-axis (10 L, 20 L, 30 L, etc.) for Fig 3B.

Response: corrected as suggested.

Reviewer #3:  The authors have utilized the hollow fiber bioreactor technology to investigate the virulence properties of Cryptosporidium parvum after 20 months within this device.
There are no major flaws in the experimental design, but the novelty is quite weak. The manuscript doesn't convince me about the outputs of the whole study and doesn't provide any novelty other than advertising the use of the HFB system.

Response: The authors feel that these comments are incorrect and do not reflect the data submitted. (i) The results go beyond previous publications by clearly demonstrating that parasites cultured in the HFB have the ability to produce an infection in calves that has the same clinical outcomes as the calf passaged isolates, and do not loose virulence properties. This cannot be demonstrated using a mouse model which we have previously published since mice do not develop diarrhea. In addition we show the genome of the in vitro cultured parasites do not demonstrate major genomic change when compared to the isolate used to initiate the HFB that was passaged for the same length of time in calves. The authors feel that these two points set this submission apart from previous ones and provide a valuable addition to the literature on this parasite.

Figure 1 is the typical figure that has been presented in all of the papers of the same group when they published HFB-related work.

Response: The authors beg to differ with the statement, the fig has not been presented in previous work, it shows the growth pattern for the parasites in the HFB over 12 months and the images demonstrate the production of oocysts which are important to the conclusions of this study. Hence, we would like to retain the image as necessary for the readers.

Supplementary figure 1; I do not understand the purpose of this experiment at all. And even if they manage to convince me about the purpose, their interpretation is also wrong since the bands (VspI samples) are not of the same size. Also, the quality of the figure is poor.
- Cryptosporidia is not a latin name and it should not be in italics.

Response: Since the fragments produced by *Ssp*l and *Vsp*l have been shown to be useful in the identification and classification of *C. parvum* isolates and produce molecular weight bands of varying size based upon the host infected [Xiao et al., Appl Env Microbiol. 1999, 65(8):3386], the authors considered that these sites may be subject to change due to in vitro culture. However, the authors agree that the genome sequencing data essentially covers this information so we have removed the Fig from the supplementary data.

Cryptosporidia is not a latin name and it should not be in italics.

Response: The italics for Cryptosporidia on page 3 (line 4) has been removed.
